# Imaging findings of superficial angiomyxoma in the eye socket

**DOI:** 10.1002/ccr3.7374

**Published:** 2023-05-18

**Authors:** Guiwu Chen, Wenqin Liu, Yongjie Cao, Xiaodong Guo, Yuhuan Xie

**Affiliations:** ^1^ Department of Ultrasound, Affiliated Dongguan Hospital Southern Medical University, Dongguan People's Hospital Dongguan China

**Keywords:** computed tomography, magnetic resonance imaging, pathology, superficial angiomyxoma, ultrasonography

## Abstract

**Key Clinical Message:**

A 23‐year‐old male with a tumor in the eye socket was characterized by multimodal images, including ultrasonography, computed tomography, and magnetic resonance imaging. After admission, surgical resection of the tumor was performed and superficial angiomyxoma was confirmed. Two years later, this tumor recurred in the same location.

**Abstract:**

Superficial angiomyxoma (SAM) is a rare benign neoplasm composed mostly of myxoid material that can affect many parts of the body in middle‐aged patients. Only a few case reports have involved imaging, which is extremely insufficient. Here, we present a case of SAM in the eye socket evaluated by imaging, including ultrasonography, computed tomography, and magnetic resonance imaging. The patient underwent surgical resection, and the diagnosis of SAM was confirmed. During the postoperative follow‐up, the tumor recurred in the same location without metastasis 2 years later.

## Graphical abstract

Superficial angiomyxoma is an uncommon, benign, multilobular neoplasm occurring mostly in middle‐aged patients. Here, we presented a case in the eye socket of a 23‐year‐old male characterized by ultrasonography, computed tomography, and magnetic resonance imaging as well as confirmed with pathology.

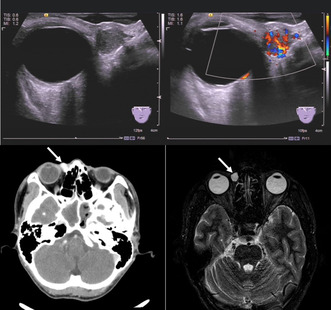

Superficial angiomyxoma (SAM) is an uncommon, benign, multilobular neoplasm composed mostly of myxoid material that shows sparse cellularity and abundant vascularization. According to the previous literature, solitary SAMs are frequent on the head or distal areas of the limbs, which are not associated with the Carney complex; multiple SAMs in patients with the Carney complex are usually located on the eyelids and in the external auditory canal.^1^ However, only a few case reports have involved imaging, and most of them are limited. Here, we report a case of SAM in the eye socket characterized by imaging, including ultrasonography, computed tomography, and magnetic resonance imaging.

A 23‐year‐old male was admitted to our hospital for the evaluation of a tumor in his right eye socket, which was present for more than 2 years. After admission, an ophthalmological examination revealed that the tumor was tough, immovable, and smooth and caused no pain. Initially, ultrasonography of the eyes revealed the tumor was located in the area of the inferior lacrimal sac (Figure 1). Next, computed tomography (Figure 2) and magnetic resonance imaging (Figure 3) of the eyes were undertaken to characterize the tumor. Furthermore, the tumor was resected by blunt dissection and pathological examination confirmed SAM without an intact capsule (Figure 4). Unfortunately, this tumor recurred in the same location without metastasis 2 years later during the postoperative follow‐up.

**FIGURE 1:**
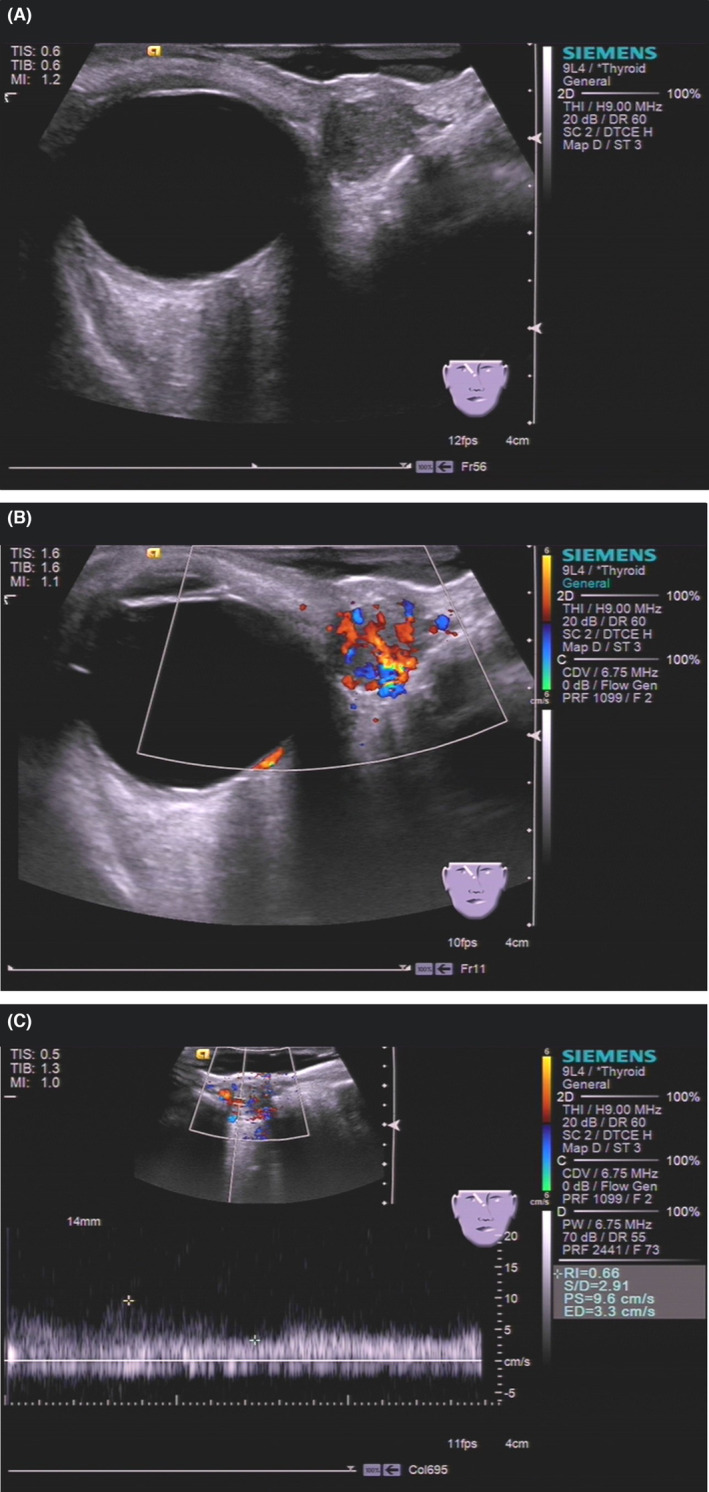
Ultrasonography of superficial angiomyxoma. (A) Grayscale ultrasound showed that the tumor was oval, defined, hypoechoic, homogeneous, and 12 mm × 10 mm in size. (B) Color Doppler flow imaging showed that multiple small and main vessels were visualized inside the tumor. (C) Pulsed‐wave Doppler showed the systolic blood flow velocity was 9.6 cm/s with a high resistance index.

**FIGURE 2:**
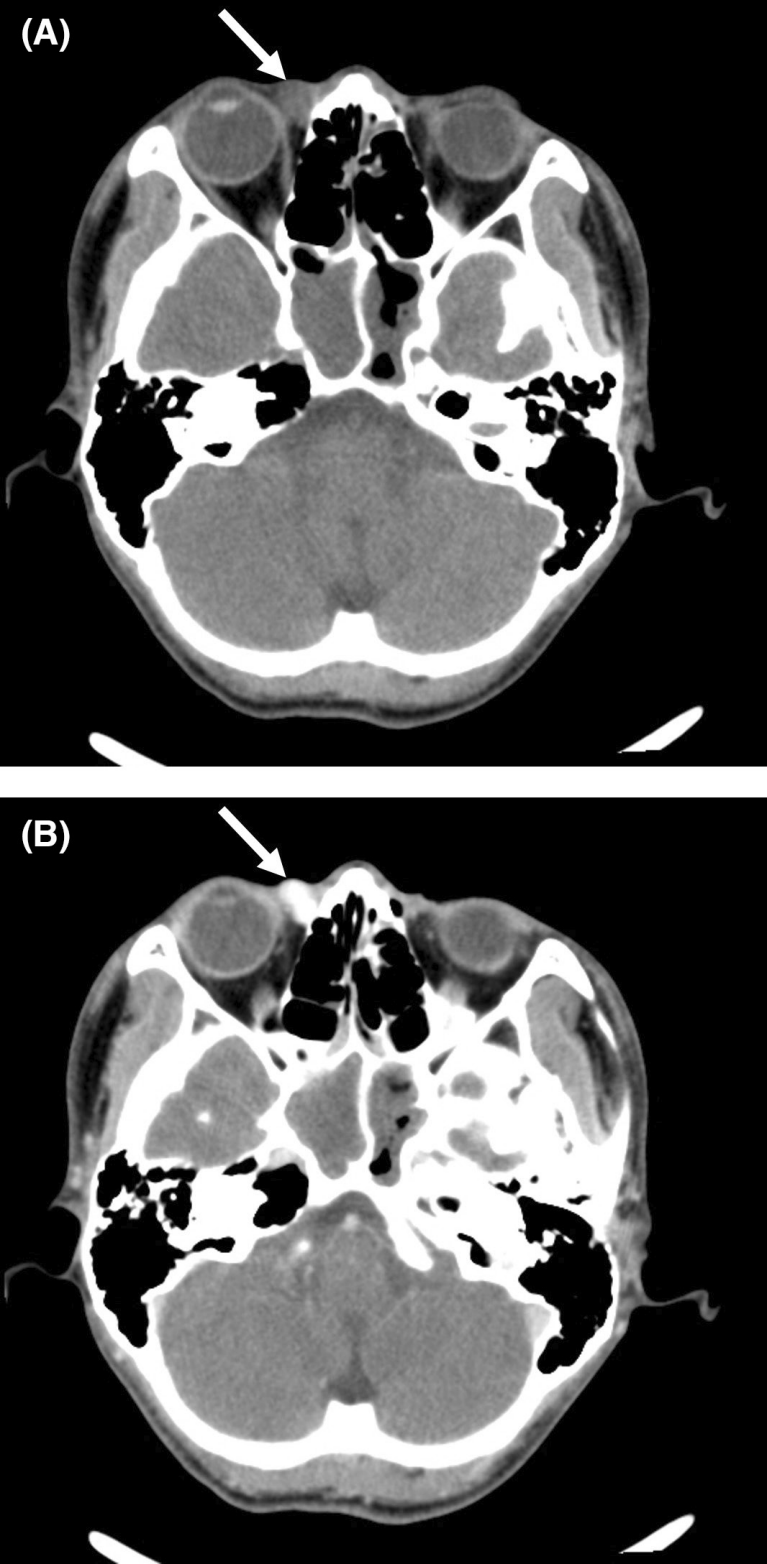
Computed tomography of superficial angiomyxoma. (A) Plain computed tomography showed that the tumor (arrow) under the medial rectus muscle of the right eye was nodular and comparatively defined with a value of 44 HU. (B) Enhanced computed tomography showed that the tumor (arrow) was obviously enhanced with a value of 157 HU–178 HU.

**FIGURE 3:**
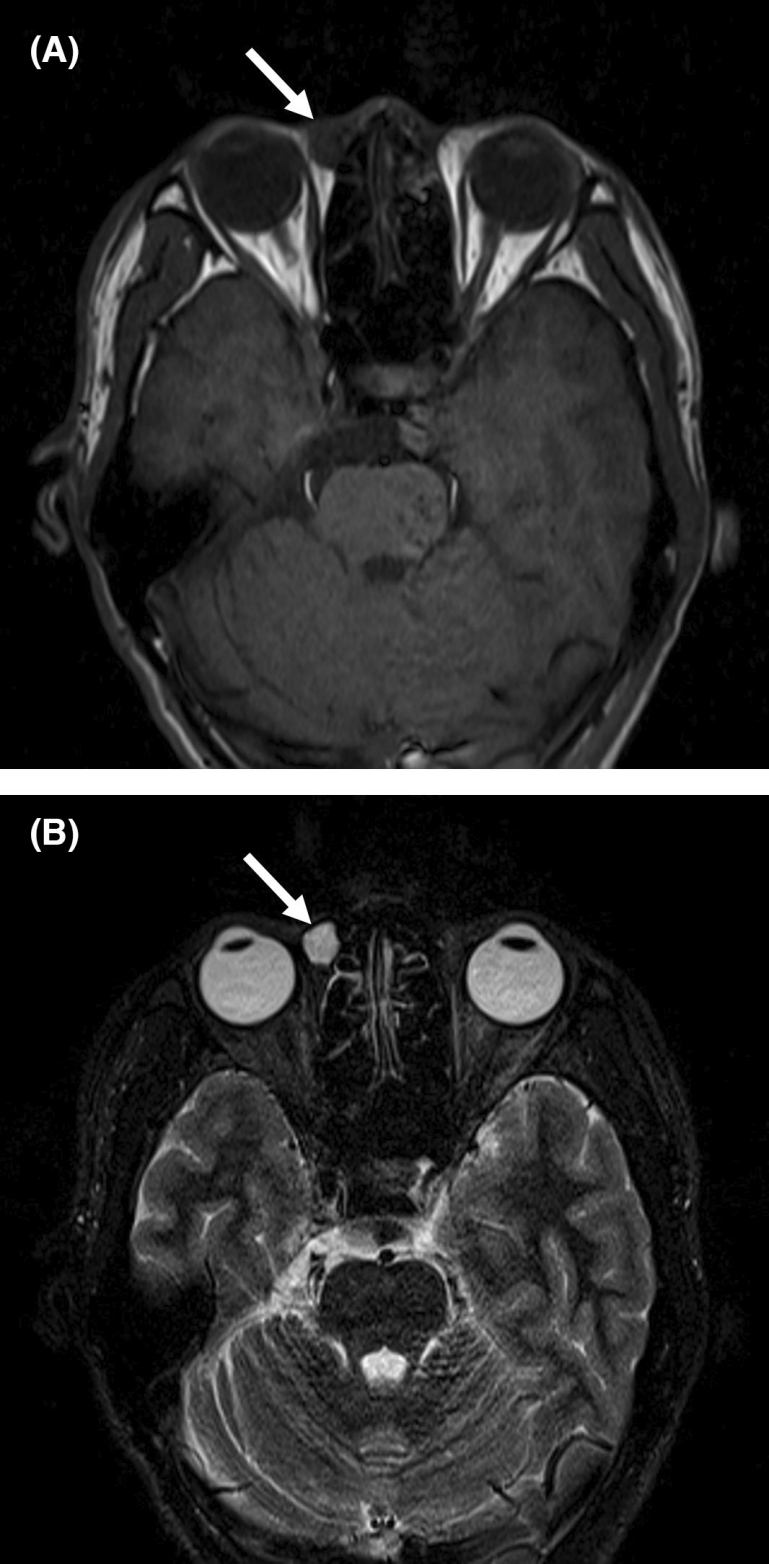
Magnetic resonance imaging of superficial angiomyxoma. (A) The tumor (arrow) near the right lacrimal sac was circular and defined on T1‐weighted images. (B) T2‐weighted images showed that the tumor (arrow) had long signals.

**FIGURE 4:**
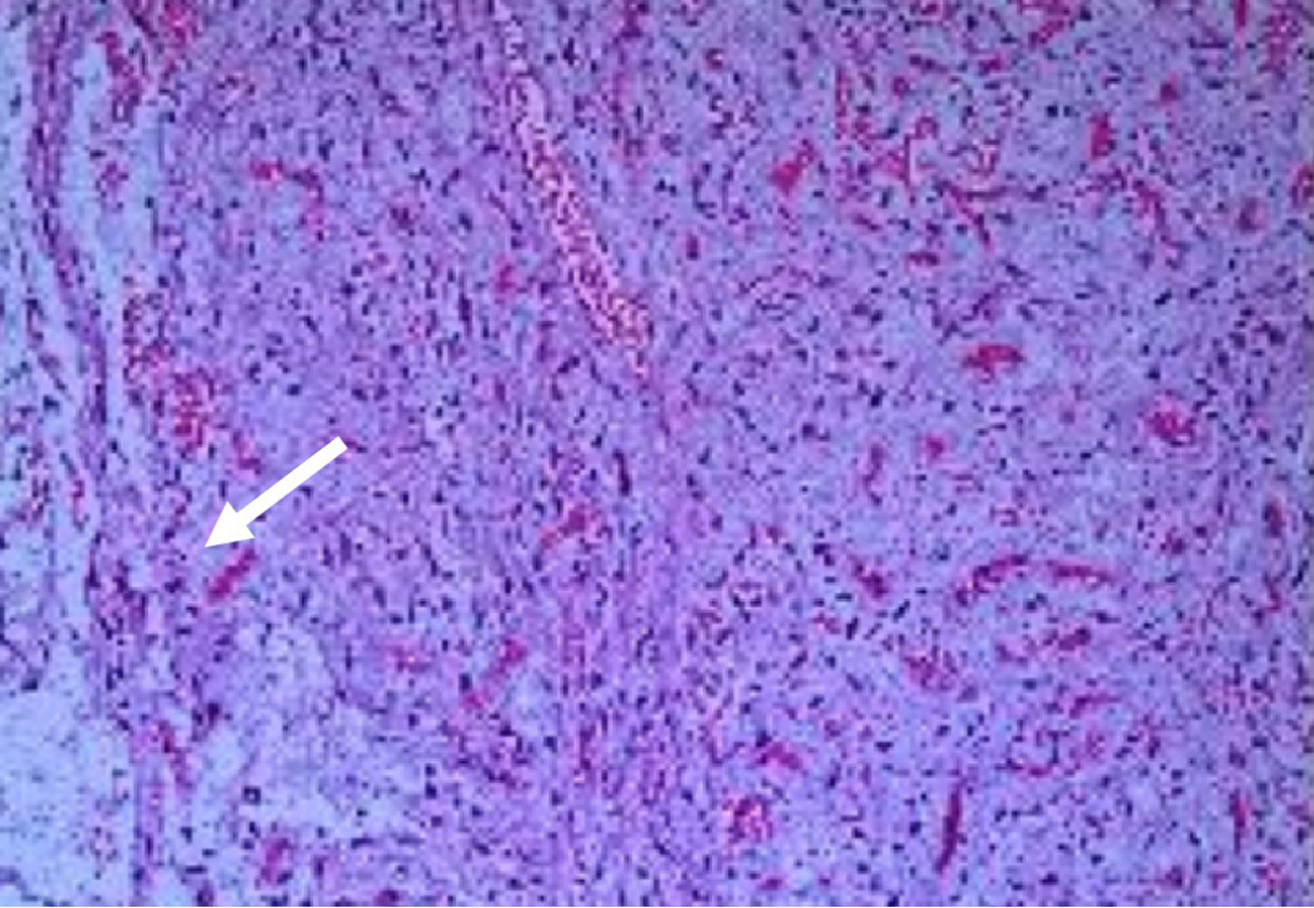
Pathology of superficial angiomyxoma. Hematoxylin–eosin staining revealed the capsule of the tumor (arrow) was broken after surgical resection, which was composed of spindle cells and stellate cells with small vessels in an abundant myxoid stroma.

Superficial angiomyxoma is an extremely rare benign tumor occurring mostly in middle‐aged patients; both males and females are equally affected. This tumor can affect many parts of the body, although the head, neck, trunk, and limbs are the most likely locations. Palpation of SAM reveals a soft, mobile, nonulcerated, and well‐defined tumor, with a range of polypoid to papulonodular appearances. On ultrasonography, SAM is a hypoechoic and heterogeneous tumor with a regular shape, well‐defined boundary, capsule, and posterior acoustic enhancement, which has a great variety of blood flow signals.^2^ Computed tomography findings of SAM show an ovoid low‐density tumor with a slight or significant enhancement. Additionally, magnetic resonance imaging has shown that SAM is a relatively well‐circumscribed tumor with hypervascularity that shows an inner homogenous high signal intensity. However, it is necessary to differentiate SAM from epidermoid cyst, neurofibroma, lymphocyst, and aggressive angiomyxoma.

Due to the rarity of SAM, it is uncommon for surgeons to accurately predict, or misdiagnose it as a common benign soft tissue tumor, and biopsies or simple excisions are normally carried out.^3^ Therefore, SAM reportedly has a high recurrence rate owing to incomplete resection while complete or wide resection could avoid this recurrence. Fortunately, recurrence tends to be local and nondestructive without distant metastases.

## AUTHOR CONTRIBUTIONS

**Guiwu Chen:** Data curation; writing – original draft; writing – review and editing. **Wenqin Liu:** Data curation; writing – original draft; writing – review and editing. **Yongjie Cao:** Investigation; validation; visualization. **Xiaodong Guo:** Data curation; validation; visualization. **Yuhuan Xie:** Project administration; supervision; writing – review and editing.

## FUNDING STATEMENT

No funding was received for this study.

## CONFLICT OF INTEREST STATEMENT

The authors declare no conflicts of interest.

## TRANSPARENCY STATEMENT

We can confirm that this manuscript is an honest, accurate, and transparent account of the case being reported and that no important aspects of the case have been omitted.

## ETHICAL STATEMENT

The corresponding author had the written consent of the patient to use the data for publication.

## CONSENT

Written informed consent was obtained from the patient to publish this report in accordance with the journal's patient consent policy.

## Data Availability

The data used to support the findings of this study are available from the corresponding author upon request.
